# Chronic Discharges from the Ear and Their Treatment

**Published:** 1898-12-17

**Authors:** Macleod Yearsley

**Affiliations:** Assistant-Surgeon to the Royal Ear Hospital; Surgeon-in-Charge of the Department for Diseases of the Throat, Nose, and Ear, the Farringdon General Dispensary; Hon. Surgeon for Diseases of the Throat and Ear, the Governesses's Home, &c.


### Hospital Clinics and Medical Progress.

### CHRONIC DISCHARGES FROM THE EAR AND THEIR TREATMENT.

By Macleod Yearsley, F.R.C.S., Assistant-Surgeon to the Royal Ear Hospital; Surgeon-in-Charge of the Department for Diseases of the Throat, Nose, and Ear, the Farringdon General Dispensary; Hon. Surgeon for Diseases of the Throat and Ear, the Governesses's Home, &c.

(*Read before the South-West London Medical Society, November 9th,* 1898.)

(*Concluded from page* 175.)

The two adjuncts to syringing are instillations and powders. The insufflation of dry powders into the ear is a method which, if not carried out with the greatest care, may result in disaster. We cannot always trust our patients, and a man whose ear has been filled with, say, powdered boric acid and allowed to depart with the strictest injunctions to return the following day, is quite capable of walking about for a week with a rocky cake of mixed powder and pus in his ear and pent-up discharge doing all sorts of damage behind it. It is always the doctor and not the patient who gets the blame in such cases, and I, therefore, advise that the dry powder method be left severely alone. Hot instillations, on the other hand, are both more manageable and more efficacious, but they require using with method. The patient should lie down upon the sound side, and the fluid, comfortably warm, poured, or instilled with a dropper, into the ear; it should then be retained for about ten minutes. If the perforation is large the fluid will reach all parts of the tympanic cavity easily, but if the perforation is a small one there may be difficulty in getting it to penetrate properly. In such cases it should be helped by pressing the tragus backwards and inwards. Instillations are best used immediately after syringing, the ear being first carefully dried by absorbent wool. Once or twice a day will be sufficient. The number of drugs which have been recommended or suggested for instillation is enormous, and I do not intend to weary you with a list of them. Those that are most useful I have divided into groups, so that we may take them with some method. Beginning with the antiseptics, these are carbolic acid, corrosive sublimate, resorcin, salicylic acid, boro-glyceride, and peroxide of hydrogen. These are the chief of them. Time will only allow me to merely indicate how most of them are used. Carbolic acid is most efficient in a weak alcoholic solution, such as one part of the acid to fifteen parts of rectified spirit and fifteen of water. It should certainly not be used any stronger than that. Perchloride of mercury may be used in either watery or alcoholic solution in the proportion of one 1·20th part in fifty parts of water, or in twenty-five parts each of water and rectified spirit. Unless the effect is good it should not be used for longer than two or three weeks. Resorcin may be used in spirit or water to the strength of 4 per cent.; salicylic acid in water (1 in 200) or spirit (2 to 5 per cent.). This drug may, however, produce a severe reaction with inflammation of the external meatus. Boro-glyceride is recommended in a solution of 10 to 50 per cent. Peroxide of hydrogen is often very efficient, and may be used in a 6 per cent. solution. Before leaving the antiseptic treatment, mention must be made of a very valuable method, especially in recent cases. The ear can stand very powerful antiseptics; indeed, its endurance in this way is often surprising, and is exceedingly useful in operations through the meatus. The method to which I allude is based upon this fact. The meatus and as much as possible of the tympanum is thoroughly purified by first syringing with 1 in 40 carbolic, carefully drying with absorbent wool, and then by swabbing out thoroughly with Lister's "strong mixture," which is a solution of 1 in 25 carbolic acid with 1·500th part of perchloride of mercury. This swabbing out must be very thoroughly done, and the meatus is then packed with double-cyanide gauze soaked in 1 in 40 carbolic and left alone for from 24 to 48 hours. It is not applicable to cases where there is any caries of the ossicles or temporal bone, or where cholesteatomata have formed, but in simple uncomplicated cases the effect is often astonishing.

We have, in the alcohol treatment, a very valuable agent, especially where antiseptic instillations fail. It consists in the instillation of warm rectified spirit once or twice a day. As, however, the pure spirit often causes severe burning at first, it should be used at the beginning diluted with an equal part of water, and then gradually increased in strength. It may be advantageously combined with antiseptics by adding to it boric acid (ʒj to ℥j), or carbolic acid (1 in 30). Should alcohol cause headache or vertigo, or should there be any caries present, it must not be employed.

The caustic treatment is one much less used than formerly. Pure chromic acid applied to small granulations is a very valuable aid to treatment, and at times nitrate of silver is found useful. The use of the latter by instillation is, however, one requiring great care, and should most certainly never be entrusted to the patient.

Astringents have of late years given way to antiseptics and alcohol. They are, however, still of value on occasion, and one of the most useful instillations I know is a mixture of six grains of sulphate of zinc with one grain of carbolic acid to half an ounce of glycerine and an ounce and a half of water, ten drops being used warm.

By means of one or other of these methods, combined with appropriate general treatment and the removal of any cause found in the nose or naso-pharynx, one should be able to cure the majority of the chronic middle-ear suppurations. There will remain, however, a few that resist treatment and require further and more vigorous means. Roughly speaking, they will be those in which there is caries, the formation of cholesteatomata, or involvement of the mastoid antrum, or in which the cause of the suppuration is tubercle. Before speaking of these, however, a few words are needed as to the treatment of those cases in which the attic is involved, either primarily or by extension. As has been said, these cases are often very tedious and obstinate in their course. Occasionally the method already described will suffice, but often they require more detailed treatment by washing out the attic daily by means of an attic syringe or cannula. Owing to the frequency with which they are complicated with caries of the head of the malleus, or of the anterior attic wall, a good deal of what has to be said regarding caries of the ossicles applies to them. When caries of the temporal bone is the cause of the obstinacy of the suppuration some operation is imperative, and delay in such cases is dangerous. It is impossible in this paper to discuss the subjective and objective symptoms of this complication or its operative treatment; suffice it to say that the nature of the discharge, which may be copious and creamy, or thin and bloody, but is always offensive, together with the changes to be seen in or around the ear, and especially the disturbances which arise when the facial nerve is involved, should lead one to suspect the nature of the case. Caries of the ossicles is a frequent cause of the resistance of chronic suppuration to treatment, and great improvement in our results has appeared since the removal of the ossicles ("ossiculectomy") has become a recognised operation in otology. The operation is undertaken to remove the cause of the discharge or to improve the hearing after the discharge has ceased. It is with the former that we have to do here. When there is a persistence of discharge with a perforation of Shrapnell's membrane, chronic attic trouble is indicated, and, when the anatomical relations of this part of the tympanum are considered, it will be seen that disease there is an hourly menace to life. The chief cause of this persistence is caries of the head of the malleus, or body of the incus, or both, or of the anterior attic wall. Nothing short of removal of the bones in question, and curetting of the bony wall will be of use, and, unless there be indications for opening the antrum, this should certainly be done before proceeding to the more grave mastoid operation. When discharge persists with a perforation in the posterior-superior segment of the membrane, the usual cause is caries of the descending process of the incus, a complication by which the attic may later become secondarily affected. Removal of the malleus, together with the remains of the incus and the membrane is indicated, and in most cases is followed by a rapid improvement. It is when the perforation is in these two situations that ossiculectomy is indicated, the caries which one sees at the tip of the handle of the malleus yielding to ordinary treatment.

It only remains to speak upon the treatment of cholesteatomata. There is but one way to deal with them—that is, removal. The method by which this is effected depends upon their position, varying according as they are in the upper or lower part of tympanum or extending into the mastoid process. In the latter case they can only be efficiently removed by a mastoid operation. When only in the tympanum, careful loosening and washing out with the intratympanic syringe may be all that is required, but often one has to remove them under general anæsthesia. When in the attic cholesteatomatous collections are often especially difficult to get away, requiring most careful washing out with a special cannula.

